# Outbreak investigation of measles in Kaduna State, Northwestern Nigeria, 2015

**DOI:** 10.11604/pamj.supp.2018.30.1.15445

**Published:** 2018-05-16

**Authors:** Aisha Ahmed Abubakar, Abdulrazaq Abdullahi Gobir, Ibrahim Ismaila Nda, Ibrahim Usman Kusfa, Babalola Obafemi, Olaniran Alabi, Meeyoung Mattie Park, Shreya Kothari, Joseph Asamoah Frimpong, Patrick Nguku

**Affiliations:** 1 Ahmadu Bello University Zaria, Nigeria; 2 Nigeria Field Epidemiology & Laboratory Training Programme; 3 Rollins School of Public Health, Emory University, Atlanta USA; 4 African Field Epidemiology Network, Accra, Ghana

**Keywords:** Outbreak investigation, measles, Nigeria

## Abstract

Sub-Saharan Africa reports repeated outbreaks of measles, a vaccine preventable disease, which is notifiable under the Integrated Disease Surveillance and Response strategy in Nigeria. Nigeria has reported several outbreaks of measles in the last three years. Poor immunization coverage and weak health systems have been related with measles. This case study is based on real events that occurred during the 2015 outbreak of measles in Kaduna state Northwestern Nigeria. This case study was based upon real events that occurred in community X in Igabi LGA of Kaduna state. However, some of the results were edited to allow the case study to be completed in a facilitated classroom session. Knowledge and practice of investigating outbreaks is a key public health function of public health workers. The purpose of this case study is to simulate outbreak investigation for teaching of postgraduate public health practitioners. The participants should have received lectures or other training on outbreak investigation without the practical experience of investigating an outbreak but are being prepared to investigate outbreaks in the field. This case study should be taken in a classroom setting and should take two hours to complete.

## How to use this Study

**General instructions:** this case study should be facilitated by one facilitator with 8-10 participants. It should be facilitated in a classroom setting. The case study should be interactive allowing the participants to learn from each other.In some aspects group discussion and role play should be used to facilitate learning.

**Audience:** residents in the Field Epidemiology & Laboratory Training Program (FELTP), Field Epidemiology Training Program (FETP), West African College of Physicians (WACP), National Postgraduate Medical College (NPMC) and Master in Public Health students.

**Prerequisites:** before using this case study, participants should have received lectures in basic epidemiology, outbreak investigation and biostatistics.

**Materials needed:** computers with MS Excel or graph paper, calculators, flip charts and markers.

**Level of training and associated public health activity:** novices who have little or no experience with outbreak investigation in a field setting.

**Time required:** 2-3 hours.

**Language:** English.

## Competing interest

The authors declare no competing interest.

## References

[1] Mobilising for Development

[2] Federal Ministry of Health Nigeria and WHO Nigeria (2012). Guidelines for measles surveillance and outbreak response Nigeria.

[3] Heymann DL (2015). Control of Communicable Disease Manual.

